# Effect of a Constant Rate Infusion of Ketamine on a Variable Rate Infusion of Xylazine in Standing Horses Undergoing Ventriculocordectomy and Laryngoplasty

**DOI:** 10.3390/vetsci13010077

**Published:** 2026-01-12

**Authors:** Francisco Medina-Bautista, Juan Morgaz, Setefilla Quirós-Carmona, María Esther Caravaca-Paredes, Rocío Navarrete-Calvo, Antonia Lucía Sánchez de Medina, Rafael Gómez-Villamandos, María del Mar Granados

**Affiliations:** Section of Anesthesiology, Department of Animal Medicine and Surgery, Veterinary Teaching Hospital, Universidad de Córdoba, 14014 Córdoba, Spain; v92moroj@uco.es (J.M.); v42quics@uco.es (S.Q.-C.); mesthercp93@gmail.com (M.E.C.-P.); v92nacar@uco.es (R.N.-C.); tsmedina189@hotmail.com (A.L.S.d.M.); pv1govir@uco.es (R.G.-V.); pv2grmam@uco.es (M.d.M.G.)

**Keywords:** ketamine, CRI, standing, horses, sedation, xylazine, VRI

## Abstract

Standing sedation in horses provides immobilization and analgesia for surgery while avoiding the high risks of general anesthesia. Ketamine at subanesthetic doses may enhance sedation and reduce xylazine requirements, but evidence in clinical settings is limited. In a randomized blinded trial, we evaluated whether adding a low-dose ketamine infusion could reduce the xylazine dose required for effective sedation during standing ventriculocordectomy and laryngoplasty. Fifty-one horses were randomly assigned to sedation with xylazine alone (SX group) or xylazine plus ketamine (KX group) in a continuous rate infusion. The ketamine group received ketamine (0.25 mg/kg intravenous (IV) bolus followed by 0.5 mg/kg/h infusion), while xylazine was administered in both groups via a titrated infusion to effect according to the Ghent Sedation Algorithm. Sedation depth, ataxia, surgical condition scores, and cardiorespiratory parameters were recorded. Data are presented as median (25th–75th percentiles) and estimated effect with 95% confidence intervals (CI). Statistical significance was set at *p* < 0.05 and at 95% CIs excluding zero. The addition of ketamine did not significantly reduce xylazine requirements (0.9 (0.7–1.3) vs. 0.8 (0.5–1.1) mg/kg/h for SX and KX, respectively; *p* = 0.139). However, horses receiving ketamine (KX) achieved deeper sedation (Estimate = 2.74; 95% CI: 0.95 to 4.63) with no differences in ataxia or surgical conditions. Cardiorespiratory variables remained stable in both groups, and no adverse events occurred. In conclusion, adding a subanesthetic ketamine infusion improved sedation depth without adverse effects but did not significantly reduce the xylazine requirement.

## Simple Summary

Performing surgery on standing horses under sedation helps avoid the high risks of general anesthesia. However, effective sedation is needed to keep the horse still and pain-free without causing harm. Xylazine is a common horse sedative, but higher doses can cause side effects like wobbliness and heart or breathing problems. This study explored whether adding a low dose of ketamine as a continuous infusion could reduce the amount of xylazine rate infusion needed and improve sedation quality during standing throat surgery. Fifty-one horses undergoing these procedures received either xylazine alone or xylazine with a low-dose ketamine infusion. The level of sedation, postural stability, ease of performing surgery, and vital parameters were recorded. Adding ketamine did not noticeably reduce the xylazine required; both groups ended up needing similar amounts. However, horses that received ketamine were slightly more deeply sedated without becoming more unsteady. Heart rate and respiratory rate remained normal in all horses, and no complications occurred. In summary, the addition of a low-dose ketamine infusion to xylazine improved sedation degree while maintaining postural stability and cardiorespiratory safety during upper way surgery.

## 1. Introduction

Standing sedation has become a cornerstone in equine practice over the past decade motivated by the high morbidity and mortality rates associated with general anesthesia in horses [1,2]. A recent large-scale study has reported a mortality rate of approximately 0.2% for standing procedures, in contrast to 1.2% for general anesthesia [3,4]. Procedures such as ventriculocordectomy and laryngoplasty involve sustained mechanical stimulation of the larynx and surrounding tissues, which may result in breakthrough responses, frequent infusion rate adjustments, or the need for supplemental α_2_-agonist boluses, potentially increasing the risk of ataxia [5].

Among the α_2_-adrenergic agonists used for this purpose, xylazine is the oldest agent in clinical use [6]. Several non-surgical experimental studies have investigated the sedative efficacy, cardiorespiratory and postural safety of xylazine in horses. Ringer et al. [7] first demonstrated a significant reduction in head height following intravenous administration, indicating an effective degree of sedation. More recently, Hopster et al. [8] reported that xylazine administered at 0.5 mg/kg followed by a CRI of 1 mg/kg/h for up to 6 h maintained a constant level of sedation without the need for dose adjustments, likely due to limited drug accumulation. Although the sedative effects and cardiopulmonary effects of xylazine are generally less pronounced and short-acting than those of romifidine, xylazine administration has been associated with a tendency toward greater postural instability and cardiopulmonary effects [9,10]. To reduce the dose of xylazine and mitigate such side effects, several adjuncts have been investigated. When it was combined with remifentanil, results have been inconsistent: while Pallarols et al. [11] found the combination provided adequate sedation and cardiorespiratory stability, Funcia et al. [12] reported adverse effects including gastrointestinal hypomotility and sporadic excitation. Ferreira et al. [13] also showed the intravenous lidocaine efficacy in enhancing sedation and analgesia but noted moderate ataxia that could compromise the safety of standing procedures. In the same way, in donkeys, xylazine combined with butorphanol provided reliable sedation, though moderate ataxia [14]. Ketamine, widely used as an anesthetic in both human and veterinary medicine, acts primarily as an N-methyl-D-aspartate (NMDA) receptor antagonist, modulating central nociceptive processing and reducing central sensitization and temporal summation of pain [15,16,17,18,19]. Ketamine has been incorporated into standing sedation CRI protocols to enhance antinociception and facilitate procedural compliance [1]. While ketamine does not consistently induce sedation in awake equids at subanesthetic doses, its antinociceptive effects have been demonstrated using nociceptive withdrawal reflex models and experimental pain paradigms in ponies and horses [20,21,22]. In anesthetised ponies, Knobloch et al. [20] demonstrated that a target-controlled infusion of ketamine achieving plasma concentrations of 1 µg/mL significantly reduced the amplitude and duration of the nociceptive withdrawal reflex, supporting its central antinociceptive activity under general anesthesia. It was shown that racemic ketamine has superior antinociceptive effects in ponies than S-ketamine, likely due to its longer duration of action [21]. However, research supporting its use in this context remains limited. At subanesthetic doses (0.4–0.8 mg/kg/h), it was well tolerated in awake horses without behavioral excitation. However, no analgesic effect was demonstrated using a modified hoof tester applied to the withers and radius [22]. Attenuation of sustained nociceptive input may reduce defensive motor response and improve surgical tolerance, thereby enhancing perceived sedation quality without requiring escalation of α_2_-adrenergic agonist doses. In clinical scenarios, ketamine has been used in combination with xylazine only in two studies, but results are controversial. Wagner et al. [23] administered ketamine at 1 mg/kg/h combined with xylazine (0.5 mg/kg bolus + 0.5 mg/kg/h), with and without butorphanol. The ketamine–xylazine combination improved tolerance to pressure stimuli as measured by algometry but failed to suppress reactions to acute nociceptive inputs such as arthrocentesis or needle pricks. Addition of butorphanol slightly enhanced pressure tolerance, but reactions to sharp pain remained. Müller et al. [24] evaluated romifidine-based standing sedation for cheek-tooth removal using fixed-dose protocols that combined romifidine either with butorphanol, midazolam, or ketamine (0.5 mg/kg followed by 1.2 mg/kg/h). Sedation quality and surgical conditions improved when ketamine was added. However, the reliance on fixed-dose regimens, administered either as boluses or constant rate infusions, limits the ability to minimize and promptly address motor instability. This highlights the potential need for objective monitoring of standing sedation quality to allow α_2_-agonist dosing to be titrated to clinical effect, particularly when combined with adjunct drugs such as ketamine, which can modify sedation characteristics [24].

The aim of this study was to evaluate the effect of adding a ketamine CRI to a xylazine variable rate infusion (VRI) in standing horses undergoing ventriculocordectomy and laryngoplasty. We hypothesized that the addition of a ketamine CRI would improve sedation quality during standing upper airway surgery by reducing the xylazine infusion rate required to achieve adequate surgical conditions and thereby limiting xylazine-associated ataxia.

## 2. Materials and Methods

The experimental protocol was approved by the local Ethics Committee for Animal Welfare of the University of Córdoba (CEBAHCV 40/2019). This study was designed and reported in accordance with the ARRIVE 2.0 guidelines [25]. Written informed consent was obtained from the owners of the horses prior to enrolment in the study.

### 2.1. Experimental Design

This prospective randomized blinded clinical trial was conducted to evaluate the incorporation of ketamine at subanesthetic doses in the standing sedation protocols in horses undergoing ventriculocordectomy and laryngoplasy. A sample size analysis was performed to determine the number of animals required to detect a clinically significant reduction of 0.32 mg/kg/h in the xylazine VRI, assuming a standard deviation of 0.4 mg/kg/h. These values were obtained from retrospective clinical records from the past five years at our hospital prior to the start of the study, in which a baseline mean xylazine dose of 1.0 mg/kg/h and a standard deviation of 0.4 mg/kg/h were recorded. In the literature, xylazine infusion rates reported in horses generally range between 0.65 and 1.1 mg/kg/h [7,9,10,13]. In this study, we chose to follow protocols using higher doses, such as those described by Fernandes de Souza et al. [13], as the intervention was performed in a clinical setting with continuous surgical stimulation, and these protocols also aligned with our institution’s average. A minimal clinically important difference of 0.32 mg/kg/h was used, consistent with that reported in a previous study [26]. It was determined that 21 horses per group were required to achieve a statistical power of 80% with a 5% significance level using a one-tailed test. Considering an expected dropout rate of 20% due to the clinical nature of the study, 26 horses were included per group. Statistical analyses were performed with the free software Jamovi (v. 2.6) and R (version 4.4.2) (Jamovi Project, Sidney, Australia). Horses were randomly assigned to one of the two treatment groups using a web-based randomization tool (http://www.random.org/, accessed on 15 December 2019). Horses in the ketamine–xylazine group (KX) received a loading dose of ketamine (0.25 mg/kg) followed by a constant rate infusion (CRI) of 0.5 mg/kg/h. Xylazine was administered as a VRI starting at 0.5 mg/kg/h after a loading dose of 0.7 mg/kg. The saline–xylazine group (SX) received the same volume and infusion rate, with saline solution replacing ketamine. All sedation procedures were performed by the same anesthesiologist (M.d.M.G.).

### 2.2. Horses

Client-owned Spanish Purebred horses requiring elective ventriculocordectomy and posterior laryngoplasty were enrolled at the Veterinary Teaching Hospital of the University of Córdoba between January 2020 and December 2023. Horses classified by the Ghent Sedation Algorithm (GSA) as “impossible to perform surgery in the standing horse, proceed to general anesthesia (GA)” were excluded from the study [27]. The horses were admitted one day prior to surgery for acclimatization and completion of preoperative evaluations. Upon admission, all horses underwent dynamic endoscopic examination, and only those diagnosed with grade III or IV recurrent laryngeal neuropathy according to the Havemeyer endoscopic laryngeal grading system requiring surgical intervention were included in the study. A full health assessment was performed, including a physical examination, complete blood count, serum biochemical analysis, and fibrinogen. Based on the presence of clinically relevant upper airway disease, all enrolled horses were classified as American Society of Anesthesiologists (ASA) physical status II.

### 2.3. Preoperative Management

The skin over the jugular vein was clipped and aseptically prepared before administering the sedative agent. A 14G catheter (Equivet, KRUUSE, Langeskov, Denmark) was placed after a subcutaneous infiltration of 4 mL mepivacaine 2% (Mepivacaína 2%, B. Braun, Barcelona, Spain). All horses received sodium penicillin at 22,000 IU/kg intravenously (IV) (Penilevel, Laboratorios Calier SA, Barcelona, Spain), gentamicin at 6.6 mg/kg IV (GentaEquine, Dechra, Barcelona, Spain), flunixin meglumine at 2.2 mg/kg IV (Meganyl^®^, Syva, León, Spain), and dexamethasone at a fixed dose of 20 mg per horse IV (Cortexonavet^®^, Syva, León, Spain), 45 min prior to sedation with xylazine.

### 2.4. Sedation, Analgesic and Monitoring Protocol

All horses included in the study received an intramuscular injection of acepromazine (Equipromazina 5 mg/mL, Labiana, Terrassa, Spain) at 0.03 mg/kg while still in their stalls, approximately 30 min prior to the administration of xylazine. Following this period, the animals were transferred to the surgical room to undergo standing procedures. Upon arrival in the operating area, a single IV bolus dose of xylazine at 0.7 mg/kg (Xilagesic 200 mg/mL, Laboratorios Calier, Barcelona, Spain) was administered, immediately followed by the initiation of the VRI of xylazine starting at a dosage of 0.5 mg/kg/h using a volumetric infusion pump (Infusomat Space, B. Braun, Melsungen, Germany). After 10 min of xylazine infusion, a bolus of morphine at 0.1 mg/kg IV (Morphine hydrochloride 20 mg/mL, B. Braun, Melsungen, Germany) was administered. Subsequently, all horses received a CRI of 100 mL over 15 min. In the SX group, the infusion consisted of 0.9% sodium chloride solution (NaCl 0.9%, B. Braun, Melsungen, Germany), whereas in the KX group it consisted of ketamine (Ketamidor 100 mg/mL, Richter Pharma, Wels, Austria) administered at a dose of 0.25 mg/kg, diluted to a final volume of 100 mL. In both groups, the infusion was delivered using an identical infusion rate (400 mL/h) during 10 min with the same infusion pump (NIKI v4 volumetric infusion pump, Grayline Medical, Norwalk, CA, USA) to ensure a constant administration time. Following this initial infusion, a CRI was initiated and maintained until the end of the procedure. In the KX group, ketamine was diluted in NaCl 0.9% to a final concentration of 0.1% and administered at a rate equivalent to 0.5 mg/kg/h administered with infusion pump (NIKI v4 volumetric infusion pump, Grayline Medical, Norwalk, CA, USA), whereas in the SX group, NaCl 0.9% was administered at the same rate as ketamine CRI rate. All infusion bags were prepared by an investigator not involved in data collection (M.E.C.-P.) based on a paper taken from a box containing 52 papers indicating the group allocation (26 papers each labelled as SX and KX). Infusion bags were visually masked using cohesive bandage (Vetrap, 3M Center, Saint Paul, MN, USA) to ensure blinding of the surgeon and the investigator responsible for outcome assessment (M.d.M.G.).

All horses received local anesthetic blocks using a 50:50 mixture of lidocaine (Lidocaína 2%, B. Braun, Barcelona, Spain) and mepivacaine (Mepivacaína 2%, B. Braun, Barcelona, Spain). Fifty mL of this mixture was splashed into the nostrils prior to endoscope insertion for the ventriculocordectomy and between 80–100 mL before the laryngoplasty, the same anesthetic combination was injected subcutaneously and infiltrated around the surgical site by the same surgeon.

Respiratory rate (RR) was determined by visual observation of thoracic excursions, heart rate (HR) by auscultation, and systolic (SAP), mean (MAP) and diastolic (DAP) arterial pressures were measured non-invasively with the cuff placed at the most proximal section of the tail and connected to a multiparametric monitor (Multiparametric monitor, B.Braun VetCare, Barcelona, Spain). The cuff width corresponded to about 40% of the circumference of the tail. Values were recorded every 10 min from the administration of xylazine until the end of the surgical procedure. Additionally, a urinary catheter was placed in all horses to facilitate urine output monitoring during the surgery.

### 2.5. Sedation, Ataxia and Surgical Condition Evaluation and Xylazine VRI Adjustment

Sedation, ataxia, and surgical conditions were monitored every 10 min throughout the procedure by a single experienced anesthesiologist (M.d.M.G.). Assessments were performed using predefined ordinal descriptors based on the GSA [27]. For each assessment time point, the evaluator selected the descriptor that best matched the horse’s clinical presentation for sedation depth, degree of ataxia and surgical conditions. These combined assessments were then used to guide stepwise adjustments of the xylazine VRI in accordance with the GSA decision pathway. If surgical conditions remained suboptimal despite adjustments in the xylazine infusion, or if the horse exhibited sudden, undesirable movements, a rescue bolus of romifidine (4 µg/kg IV) was administered given its lower ataxic effect. The number of romifidine boli required per horse was recorded. All surgical procedures were performed by the same experienced surgeon.

### 2.6. Statistical Analysis

Statistical analyses were conducted using the free software Jamovi (version 2.6; The Jamovi Project, Sydney, Australia) and R (version 4.4.2; R Foundation for Statistical Computing, Vienna, Austria). The normality of residuals for quantitative variables was assessed using the Shapiro–Wilk test. Surgical times between groups were compared using the Mann–Whitney U test. A generalized linear mixed model (GLMM) with a Gamma distribution and log link function was used to analyze the effect of the fixed factor *Protocol* on quantitative outcomes, while accounting for the random effect of individual animal. The proportional odds assumption for ordinal variables was evaluated using the Brant test (via the *brant* package). Due to assumption violations and the need to include random effects, a Bayesian cumulative ordinal regression model was fitted. This model used a logit link and flexible thresholds, including *Protocol* and *Xylazine dose* as predictors. Random intercepts for animal and random slopes for *Xylazine dose* were included to account for inter-individual variability. Estimation was performed using Markov Chain Monte Carlo sampling with the No-U-Turn Sampler (NUTS) algorithm, implemented via the *brms* package. Four chains of 4000 iterations each were run to ensure convergence. A Bonferroni-adjusted post hoc test was performed when necessary to account for multiple comparisons. A chi-square test was used to compare the frequency of romifidine bolus administration between groups. Quantitative variables are reported as median (25th–75th percentiles), categorical variables as percentages, and ordinal variables as median (25th–75th percentiles), and estimated effect and 95% confidence intervals (CI). Statistical significance was set at *p* < 0.05 and at 95% CIs excluding zero.

## 3. Results

### 3.1. Sample Characteristics and Intraoperative Monitoring

A total of 51 horses were included in the study: 25 in the SX group and 26 in the KX group. Although the initial design included 26 animals per group, one horse in SX group had to be excluded due to an issue unrelated to the study. Age was 5 (3–10) and 5 (2–9) years in the SX and KX groups, respectively. Body weight was 560 (430–655) and 585 (485–670) kg in the SX and KX groups, respectively. The SX group comprised 23 males and 2 females, whereas the KX included 24 males and 2 females. Breed distribution in SX group was: 18 Pure Spanish Horses, 3 crossbreeds, 1 Lusitanian, 1 KWPN, and 1 Warmblood horses; In the KX group was: 20 Pure Spanish Horses, 2 crossbreeds, 1 Lusitanian, 1 KWPN, and 2 Warmblood horses. Median (25th–75th percentiles) surgical duration was 70 (30–120) minutes in SX and 70 (30–110) minutes in KX, with no significant difference between groups (*p* = 0.215). Median (25th-75th percentiles) values of the quantitative variables are shown in Table 1. No significant differences were observed between groups for HR (*p* = 0.679), RR (*p* = 0.588), SAP (*p* = 0.714), MAP (*p* = 0.780), or DAP (*p* = 0.654). The median (25th–75th percentiles) for the ordinal variables, ataxia, sedation and surgical conditions are represented in Table 2.

### 3.2. Xylazine Requirements and Ataxia

Likewise, no significant difference was found in the total xylazine dose administered (*p* = 0.139). The VRI (mg/kg/h) was 0.9 (0.7–1.3) and 0.8 (0.5–1.1) for SX and KX, respectively. No significant effects of Protocol or Xylazine dose were observed on ataxia scores, and no interaction between these factors was detected (Table 3). However, marked inter-individual variability was identified in ataxia response to xylazine, with a strong negative correlation (r = −0.90), indicating that horses with lower initial ataxia tended to exhibit a greater response to increasing xylazine dose.

### 3.3. Quality of Sedation

Protocol had a significant positive effect on sedation (Estimate = 2.74; 95% CI: 0.95–4.63), suggesting that horses receiving ketamine achieved higher sedation scores than controls. Xylazine dose also had a significant effect on sedation (Estimate = 3.00; 95% CI: 0.13–5.93), with deeper sedation associated with higher doses (Table 4). While the interaction between Protocol and Xylazine dose was not statistically significant, the estimate approached the threshold, so a relevant interaction cannot be definitively excluded. A moderate negative correlation (r = −0.73) was found between baseline sedation and responsiveness to xylazine, suggesting that horses with lower initial sedation responded more markedly to xylazine dose increases.

Romifidine administration was required in five horses in the control group, with a total of 10 rescue boluses administered. Of these five horses, one required a single bolus, three required two boluses each, and one horse required three boluses. Regarding the surgical stimuli triggering rescue analgesia, three boluses were administered in response to laser application during ventriculocordectomy, three during skin incision, three during dissection of the muscular plane, and one during local anesthetic injection. In the ketamine group, romifidine administration was required in seven horses, also resulting in a total of 10 rescue boluses. Of these seven horses, four required a single bolus, whereas the remaining three horses required two boluses each. In this group, five boluses were administered during laser application, two during introduction of the endoscope through the nostril, one during passage of the suture through the laryngeal cartilage, one during skin incision, and one during dissection of the muscular plane. No significant difference was found between groups in the number of horses requiring romifidine (*p* = 0.560) or in the number of boluses administered (*p* = 0.982).

### 3.4. Surgical Conditions

Regarding surgical conditions, no significant effect of Protocol, Xylazine dose, or their interaction were found. Xylazine dose showed a trend toward reducing surgical interference (Estimate = −1.53; 95% CI: −3.28 to 0.15), but this did not reach statistical significance (Table 5). Variability was observed between horses in both baseline response and dose-dependent responses, with no consistent correlation between these effects.

All ordinal models demonstrated good convergence, with R-hat values of 1.00 and sufficiently high effective sample sizes, confirming the reliability of parameter estimates.

## 4. Discussion

This study aimed to evaluate whether adding ketamine would allow for xylazine dose-sparing effects minimizing ataxia while improving quality of sedation and surgical conditions standing procedures in horses. The initial hypothesis was not confirmed. No significant differences were observed between groups in the total xylazine dose administered, sedation scores, ataxia degrees, or surgical conditions. However, deeper levels of sedation were achieved in the KX group. Variability was observed between horses in both baseline sedation and their dose-dependent responses, with no consistent correlation between these effects. In contrast, despite increasing the xylazine dose tended to decrease surgical interference, a marked inter-individual variability in ataxia response to xylazine was identified. A strong negative correlation (r = −0.90) indicated that horses with lower initial ataxia tended to exhibit a greater increase in ataxia with increasing xylazine doses. This substantial inter-individual variability, combined with the ordinal nature of the outcome variables and the presence of repeated measurements within the same subjects, necessitated a statistical approach capable of handling complex data structures. In such settings, the assumptions required for conventional ordinal logistic regression—particularly the proportional odds assumption—are frequently violated. Accordingly, a Bayesian mixed ordinal model was used in the present study, as it appropriately accounts for the ordinal scale of the outcomes, incorporates within-animal correlation through random effects, and does not rely on the proportional odds assumption, which was violated in this dataset [28].

A previous study has suggested that the coadministration of ketamine may reduce the need for α_2_-adrenergic agonists to achieve adequate sedation in standing horses [26]. When CRIs of morphine (0.1 mg/kg/h) and ketamine (1.2 mg/kg/h) were added to a xylazine VRI, a xylazine infusion rate of 0.4 mg/kg/h was sufficient to maintain adequate sedation and analgesia for carotid translocation—a moderately invasive standing procedure—compared to the 0.65 mg/kg/h frequently reported in the literature [7,9,10]. In contrast, in our study, the xylazine requirements were considerably higher—0.9 (0.5–1.1) mg/kg/h—to achieve similar clinical conditions. This discrepancy may be partially explained by differences in the surgical procedures; although ventriculocordectomy is relatively short and minimally invasive, laryngoplasty is more prolonged and technically demanding, likely requiring deeper and more sustained sedation [5]. Besides, a lower ketamine dose rate was used in the present study, limiting direct comparability with previously reported results [26].

The key mechanism supporting the inclusion of ketamine in standing sedation protocols lies in its potential to modulate nociception. Ketamine acts as an NMDA receptor antagonist, a mechanism known to reduce central sensitization and pain perception [29,30,31]. Peterbauer et al. [21], using a nociceptive withdrawal reflex (NWR) model, showed that a bolus of 0.6 mg/kg followed by a CRI of 1.2 mg/kg/h of racemic ketamine significantly suppressed the NWR, with arterial concentrations ranging between 20 and 25 ng/mL. Wagner et al. [23] also found that combining ketamine with xylazine and butorphanol improved tolerance to deep pressure stimuli (e.g., scapular algometry) and facilitated dental procedures in horses, although responses to acute, superficial pain like needle pricks were increased. These findings suggest that ketamine may enhance tolerance to sustained or deep nociceptive input—likely encountered in procedures such as ventriculocordectomy or laryngoplasty—while its effects on acute, sharp stimuli remain inconsistent.

As in small animal practice [32], therapeutic plasma concentrations of ketamine used in horses are frequently extrapolated from human medicine, where analgesic effects are typically reported at plasma levels of approximately 200–800 ng/mL [33,34]. However, attempts to define effective plasma concentrations in horses using pharmacokinetic–pharmacodynamic (PK/PD) approaches have yielded inconsistent results. Fielding et al. [22] reported behavioural excitation at infusion rates of 1.6 mg/kg/h and subsequently evaluated lower rates (0.4–0.8 mg/kg/h), achieving plasma concentrations of 67–137 ng/mL without demonstrable antinociceptive effects using a hoof tester. In contrast, Lankveld et al. [35] employed higher initial infusion rates (up to 4.8 mg/kg/h), stabilising at 1.5 mg/kg/h aiming to achieve subanesthetic plasma concentrations of approximately 0.8–1 µg/mL [36]; however, the measured mean plasma concentrations reported were approximately 235 ng/mL. Nevertheless, this protocol was associated with increased cardiovascular stimulation and behavioural alterations. Conversely, markedly lower arterial concentrations (≈16.7–25 ng/mL) have been reported following ketamine loading doses and CRIs of 0.6 mg/kg and 1.2 mg/kg/h, respectively, demonstrating antinociceptive effects using nociceptive withdrawal reflex models or clinical dental procedures [21,24,37]. Under general anesthesia, Knobloch et al. [20] achieved significant suppression of the nociceptive withdrawal reflex using target-controlled infusion of S-ketamine at 1000 ng/mL, likely corresponding to racemic ketamine plasma concentrations of ~2000 ng/mL based on the two-fold higher analgesic potency of the S-enantiomer [30]. These findings highlight substantial variability in reported plasma concentrations associated with antinociceptive or behavioural effects, reflecting differences in species, experimental models, sampling methodology, and physiological state, and underscore the absence of a clearly defined plasma–effect relationship for ketamine in conscious or sedated horses.

In our study, a loading dose of 0.25 mg/kg followed by a CRI of 0.5 mg/kg/h was ultimately selected after preliminary trials with higher doses led to undesirable effects. Initially, a loading dose of ketamine (0.5 mg/kg) over 5 min was administered; however, horses developed noticeable ataxia shortly after infusion onset. Considering these observations, the ketamine loading dose was reduced to 0.25 mg/kg and administered over 15 min to minimize motor instability and improve postural control during standing sedation.

The final protocol was well tolerated by all horses, and no adverse events were recorded during the bolus administration or throughout the entire procedures. A limitation of the present study is the absence of plasma ketamine concentration measurements. Without pharmacokinetic data, comparisons between dosing regimens and plasma concentrations reported in previous studies remain highly speculative. Although estimated concentrations may provide contextual insight, they rely on assumptions regarding drug clearance and distribution that may not hold under conditions of deep sedation and multimodal drug administration, where physiological status and drug–drug interactions could alter ketamine disposition. Using a one-compartment pharmacokinetic model described by Maitre and Schafer [38] and a clearance value reported in sedated horses by Lankveld et al. [35], the expected steady-state plasma concentration for the dosing regimen used in the present study would be approximately 157 ng/mL. Although this estimated value was below the nociceptive range of 200 ng/mL, it should be interpreted with caution, as it is not supported by direct measurements and may not accurately reflect true plasma concentrations in the clinical setting studied.

In Lankveld et al. [35]’s study, horses exhibited significant increases in RR, HR, and arterial blood pressure, accompanied by mild behavioural excitation such as weight shifting and muscle twitching. Increased alertness observed in several horses during prolonged infusions suggests enhanced central nervous system activity and indicates caution at higher infusion rates. In contrast, no such physiological stimulation was observed in our study, which may be attributed to our multimodal protocol. Marked inter-individual variability was observed in the ataxic response to xylazine in the present study. The strong negative correlation (r = −0.90) between baseline ataxia and subsequent dose-related changes indicates that horses with lower initial ataxia tended to develop more pronounced ataxia as xylazine doses increased, potentially related to differences in receptor expression, central nervous system responsiveness, or downstream signalling pathways. Although such mechanisms cannot be directly assessed in a clinical study, inter-individual differences in α_2_-agonist effects on sedation and motor control have been previously described in horses [9]. An alternative explanation is that horses presenting with minimal baseline ataxia may have tolerated higher incremental increases in xylazine infusion rates before reaching clinically unacceptable instability, thereby unmasking a steeper dose–response relationship. In contrast, horses exhibiting early ataxia may have required more conservative dose adjustments, resulting in a flatter apparent response. From a clinical perspective, this observation underscores the importance of individualised titration of α_2_-agonists during standing sedation. Horses that appear initially stable should not be assumed to tolerate further dose escalation without risk, and close monitoring of postural stability remains essential regardless of baseline presentation. These findings support the use of variable rate infusion protocols guided by repeated clinical assessment rather than fixed dosing strategies.

Previous studies have reported inconsistent effects of ketamine on postural stability. Benredouane et al. [26] found that the addition of ketamine or butorphanol to xylazine did not significantly affect ataxia scores, whereas Peterbauer et al. [21] described transient signs of ataxia and dysperception immediately after ketamine administration. Similar behavioural alterations associated with NMDA receptor antagonism have been reported in humans [39,40]. These behavioural responses have been described as dose-dependent, coinciding with peak ketamine plasma concentrations in horses [35]. Wagner et al. [23] reported that low-dose ketamine combined with xylazine and butorphanol may induce ataxia. However, when compared with other multimodal protocols, romifidine–ketamine resulted in less ataxia than romifidine alone or romifidine–midazolam, the latter producing the greatest motor instability [24]. Overall, these findings are consistent with our results and suggest that subanesthetic ketamine may limit postural instability.

Xylazine dose had a significant effect on sedation scores (Estimate = 3.00; 95% CI: 0.13–5.93), with greater sedation observed at higher doses. In both groups, high levels of sedation were achieved. It is unlikely that ketamine alone contributed significantly to sedation, as its sedative properties have not been consistently reported in the literature, even at higher doses [21,22,35,37]. Therefore, the deeper sedation observed in the ketamine group is unlikely to be solely attributed to the increased dose of the α_2_-adrenergic agonist, as xylazine requirements did not significantly differ between groups. Several alternative explanations may account for this finding. Firstly, ketamine may exert an additive or potentiating effect when combined with α_2_-agonists, enhancing overall sedation quality without directly acting as a primary sedative. Secondly, modulation of nociceptive input by ketamine may have reduced behavioural responses to surgical stimulation, indirectly influencing sedation scoring. Finally, although assessors were blinded, sedation scoring remains partially subjective, and subtle behavioural differences may have contributed to the observed group differences. In a similar context, Müller et al. [24] demonstrated that horses receiving romifidine combined with ketamine achieved better sedation quality and required fewer rescue boluses compared to those treated with romifidine alone. Although in our study the difference in xylazine consumption was not statistically significant, the consistently higher sedation scores in the KX group support the hypothesis of an additive or potentiating effect. Furthermore, despite the total number of rescue romifidine boluses did not differ between groups, differences were observed in the surgical stages triggering their administration. In the xylazine-only group, rescue boluses were more commonly required during ventriculocordectomy-related stimulation, whereas fewer boluses were administered during these stages in the ketamine–xylazine group. This observation may be related to the antinociceptive properties of ketamine, which could have attenuated nociceptive input associated with surgical tissue manipulation.

In our study, surgical conditions were evaluated during ventriculocordectomy in horses with unilateral laryngeal paralysis, a procedure that differs markedly in nature and invasiveness from those evaluated in previous reports. Müller et al. [24] assessed sedation and extraction quality during cheek tooth removal, while Benredouane et al. [26] studied horses undergoing carotid artery translocation. Both procedures involve prolonged oral or cervical manipulation and typically require less sedation than upper airway surgeries. Despite these differences, the combination of xylazine and ketamine in our protocol resulted in satisfactory surgical conditions in all cases, with no need to convert to general anesthesia even in horses with nervous temperament. In the Müller et al. [24] study, sedation with romifidine alone was insufficient for dental procedures, and the addition of ketamine improved overall surgical conditions and reduced the need for supplemental sedative boluses—similar to the trend observed in our study. These findings suggest that, even in procedures with differing stimulation profiles [22], ketamine may contribute to improving surgical compliance when integrated into multimodal sedation strategies.

Regarding cardiorespiratory safety, no significant differences were observed between groups in any of the variables evaluated (Table 1). This indicates that the addition of ketamine to xylazine did not negatively affect cardiovascular function under the conditions of this study. These results suggest that subanesthetic doses of ketamine can be safely incorporated into standing sedation protocols without compromising cardiopulmonary stability, in agreement with previous reports describing minimal cardiovascular effects with faster infusion rates [31,35]. However, future investigations should aim to combine controlled dose-titration protocols with objective nociceptive models and concurrent pharmacokinetic–pharmacodynamic analysis. Such approaches would allow a more robust characterisation of the relationship between ketamine dose, plasma concentration, antinociceptive effects, and motor stability, thereby supporting more evidence-based integration of ketamine into standing sedation protocols in horses.

## 5. Conclusions

The co-administration of a subanesthetic ketamine constant rate infusion (CRI) achieved deeper sedation, suggesting a potential additive effect, although not sufficient to achieve dose sparing of xylazine in standing horses undergoing ventriculocordectomy and laryngoplasty. Ataxia responses showed marked inter-individual variability, and it were more closely associated with xylazine dosing than with ketamine. Importantly, cardiorespiratory parameters remained stable across both groups, and no adverse events were recorded, supporting the safety of this protocol in a clinical setting. Although ketamine did not demonstrate clear dose-sparing benefits at 0.5 mg/kg/h following a bolus of 0.25 mg/kg, its inclusion in multimodal sedation protocols may still enhance tolerance to sustained nociceptive stimulation, warranting further investigation in different surgical contexts.

## Figures and Tables

**Table 1:** Continuous variables of the study expressed as median (25th–75th percentiles).

	Control Group (SX)	Ketamine Group (KX)	*p* Value
**HR** (beats per min)	32 (30–36)	32 (28–35)	0.679
**RR** (breaths per min)	9 (8–12)	10 (8–12)	0.588
**SAP** (mmHg)	121 (107–134)	112 (101–125)	0.714
**MAP** (mmHg)	88 (79–101)	86 (77–95)	0.780
**DAP** (mmHg)	74 (65–87)	73 (63–81)	0.654
**Dose of xylazine** (mg/kg/h)	0.9 (0.7–1.3)	0.8 (0.5–1.1)	0.139

HR: heart rate; RR: respiratory rate; SAP: systolic arterial pressure; MAP: mean arterial pressure; DAP: diastolic arterial pressure.

**Table 2:** Ordinal variables of the study expressed as median (25th–75th percentiles).

	Control Group (SX)	Ketamine Group (KX)
**Ataxia**	1 (1–2)	2 (1–2)
**Sedation**	3 (3–4)	4 (3–4)
**Surgery**	4 (3–4)	4 (3–4)

**Table 3:** Parameter estimates for Bayesian Ordinal Mixed Model Analysis of Ataxia severity.

	Estimate	Lower 95% CI	Upper 95% CI	R-Hat	ESS
**Thresholds**					
No ataxia—Mild ataxia	1.80	−1.38	4.97	1.00	3721
Mild ataxia—Marked ataxia	4.09	0.90	7.27	1.00	3724
Marked ataxia—Very Pronounced ataxia	6.54	3.32	9.81	1.00	3799
**Fixed Effects**					
Protocol	1.26	−0.78	3.27	1.00	3395
Xylazine dose	1.07	−1.76	3.91	1.00	3675
Protocol × Xylazine dose	−0.87	−2.70	0.96	1.00	3461
**Random Effects**					
Sd (Horse)	2.99	2.20 *	3.96 *	1.00	3797
Sd (Xylazine dose)	2.46	1.64 *	3.49 *	1.00	2937
Correlation (Horse, Xylazine dose)	−0.90	−0.96 *	−0.79 *	1.00	4518

* Significant statistical effect. It refers to effects in whose interval value 0 does not appear; R-hat = Potential Scale Reduction Factor. The closer the value is to 1.00 the more stable the model is; ESS = Effective Sample Size. Higher values (>1000) results in greater model stability; R-hat and ESS show that the Bayesian Ordinal Mixed Model Analysis on Ataxia severity was stable and reliable.

**Table 4:** Parameter estimates for Bayesian Ordinal Mixed Model Analysis of Sedation degree.

	Estimate	Lower 95% CI	Upper 95% CI	R-Hat	ESS
**Thresholds**					
No sedation—Mild sedation	−3.73	−7.35	−0.37	1.00	6613
Mild sedation—Good sedation	1.76	−1.03	4.66	1.00	5403
Good sedation—Marked sedation	4.92	2.13	7.87	1.00	5395
**Fixed Effects**					
Protocol	2.74	0.95 *	4.63 *	1.00	5157
Xylazine dose	3.00	0.13 *	5.93 *	1.00	6647
Protocol × Xylazine dose	−1.83	−3.80	0.05	1.00	4576
**Random Effects**					
Sd (Horse)	2.49	1.70 *	3.45 *	1.00	4605
Sd (Xylazine dose)	2.36	1.46 *	3.41 *	1.00	2138
Correlation (Horse, Xylazine dose)	−0.73	−0.90 *	−0.43 *	1.00	3018

* Significant statistical effect. It refers to effects in whose interval value 0 does not appear; R-hat = Potential Scale Reduction Factor. The closer the value is to 1.00 the more stable the model is; ESS = Effective Sample Size. Higher values (>1000) results in greater model stability; R-hat and ESS show that the Bayesian Ordinal Mixed Model Analysis on Sedation degree was stable and reliable.

**Table 5:** Parameter estimates for Bayesian Ordinal Mixed Model Analysis of Interference on surgery.

	Estimate	Lower 95% CI	Upper 95% CI	R-Hat	ESS
**Thresholds**					
Moderate interference—Acceptable interference	−3.21	−5.11	−1.36	1.00	5316
Acceptable interference—No interference	−1.82	−3.72	0.02	1.00	5305
**Fixed Effects**					
Protocol	−0.14	−1.32	1.04	1.00	5107
Xylazine dose	−1.53	−3.28	0.15	1.00	4947
Protocol × Xylazine dose	0.42	−0.69	1.54	1.00	2481
**Random Effects**					
Sd (Horse)	1.03	0.38 *	1.93 *	1.00	1958
Sd (Xylazine dose)	0.62	0.03 *	1.59 *	1.01	1024
Correlation	−0.45	−0.95	0.81	1.00	2481

* Significant statistical effect. It refers to effects in whose interval value 0 does not appear; R-hat = Potential Scale Reduction Factor. The closer the value is to 1.00 the more stable the model is; ESS = Effective Sample Size. Higher values (>1000) results in greater model stability; R-hat and ESS show that the Bayesian Ordinal Mixed Model Analysis on Surgery was stable and reliable.

## Data Availability

The data presented in this study are available in KetamineXylazineVRI at https://github.com/v52mebaf/KetamineXylazineVRI, accessed on 1 November 2025, reference number N/A. These data were derived from the following resources available in the public domain: GitHub repository (https://github.com/v52mebaf/KetamineXylazineVRI, accessed on 1 November 2025).

## Abbreviations

The following abbreviations are used in this manuscript:

CI	Confidence interval
CRI	Constant rate infusion
DAP	Diastolic arterial pressure
GLMM	Generalized linear mixed model
GSA	Ghent Sedation Algorithm
HR	Heart rate
IV	Intravenous
KX	Ketamine–xylazine
MAP	Mean arterial pressure
NWR	Nociceptive withdrawal reflex
RR	Respiratory rate
SX	Saline-xylazine
SAP	Systolic arterial pressure
VRI	Variable constant rate
